# Processing of Polyester-Urethane Filament and Characterization of FFF 3D Printed Elastic Porous Structures with Potential in Cancellous Bone Tissue Engineering

**DOI:** 10.3390/ma13194457

**Published:** 2020-10-08

**Authors:** Agnieszka Haryńska, Iga Carayon, Paulina Kosmela, Anna Brillowska-Dąbrowska, Marcin Łapiński, Justyna Kucińska-Lipka, Helena Janik

**Affiliations:** 1 Department of Polymers Technology, Faculty of Chemistry, Gdansk University of Technology (GUT), Narutowicza Street 11/12, 80-233 Gdansk, Poland; iga.carayon@pg.edu.pl (I.C.); paulina.kosmela@pg.edu.pl (P.K.); juskucin@pg.edu.pl (J.K.-L.); heljanik@pg.edu.pl (H.J.); 2 Department of Molecular Biotechnology and Microbiology, Faculty of Chemistry, Gdansk University of Technology (GUT), Narutowicza Street 11/12, 80-233 Gdansk, Poland; 3 Institute of Nanotechnology and Materials Science, Faculty of Applied Physics and Mathematics, Gdansk University of Technology (GUT), Narutowicza Street 11/12, 80-233 Gdansk, Poland; marcin.lapinski@pg.edu.pl

**Keywords:** filament formation, Fused Filament Fabrication, 3D printing, thermoplastic polyurethane, tissue scaffolds, material characterization

## Abstract

This paper addresses the potential of self-made polyester-urethane filament as a candidate for Fused Filament Fabrication (FFF)-based 3D printing (3DP) in medical applications. Since the industry does not provide many ready-made solutions of medical-grade polyurethane filaments, we undertook research aimed at presenting the process of thermoplastic polyurethane (TPU) filament formation, detailed characteristics, and 3DP of specially designed elastic porous structures as candidates in cancellous tissue engineering. Additionally, we examined whether 3D printing affects the structure and thermal stability of the filament. According to the obtained results, the processing parameters leading to the formation of high-quality TPU filament (TPU_F) were captured. The results showed that TPU_F remains stable under the FFF 3DP conditions. The series of in vitro studies involving long- and short-term degradation (0.1 M phosphate-buffered saline (PBS); 5 M sodium hydroxide (NaOH)), cytotoxicity (ISO 10993:5) and bioactivity (simulated body fluid (SBF) incubation), showed that TPU printouts possessing degradability of long-term degradable tissue constructs, are biocompatible and susceptible to mineralization in terms of hydroxyapatite (HAp) formation during SBF exposure. The formation of HAp on the surface of the specially designed porous tissue structures (PTS) was confirmed by scanning electron microscope (SEM) and energy-dispersive X-ray spectroscopy (EDS) studies. The compression test of PTS showed that the samples were strengthened due to SBF exposure and deposited HAp on their surface. Moreover, the determined values of the tensile strength (~30 MPa), Young’s modulus (~0.2 GPa), and compression strength (~1.1 MPa) allowed pre-consideration of TPU_F for FFF 3DP of cancellous bone tissue structures.

## 1. Introduction

For years, polyurethanes have been successfully used as materials in biomedical applications. Their complex chemical structure and possibility of modification enable the properties to be adjusted to the desired needs. Polyurethanes are widely utilized in drug delivery systems, vascular and cardiac surgery or tissue engineering, thus novel and advanced methods of their processing are being constantly developed. Recently, 3D printing (3DP) has become one of the most desired methods of product fabrication for some medical applications [1]. This is most likely due to the design freedom combined with the possible direct use of DICOM (Digital Imaging and Communications in Medicine) files, which allows for the production of patient-matched and complex 3D medical structures [2,3]. Medicine uses many different techniques of 3DP, including SLS (Selective Laser Sintering), bioprinting or PJ (PolyJet). However, polyurethanes are most often applied with SLA (Stereolithography), bioplotting or FFF (Fused Filament Fabrication) 3DP techniques [4]. FFF is one of the most common 3D printer types due to its relatively low purchase, maintenance, and feedstock costs and high availability. It uses thermoplastic-based materials in the form of fiber with a constant diameter (filament) and operates a mini-heated extruder where plastification of the polymer takes place. Such plasticized material is deposited on the platform following the loaded design path, forming the desired object.

The unique properties of polyurethanes, including flexibility, biocompatibility, hemocompatibility or degradability are highly desirable features in tissue engineering applications. Therefore, research has been undertaken on the use of thermoplastic polyurethanes (TPU) in FFF 3DP of porous tissue structures [5,6,7]. A review of works related to the polyurethane-based elastic tissue constructs formed via FFF 3DP is presented for example in Przybytek et al. [8]. Nevertheless, none of these works describes in detail the TPU filament formation process and its characteristics. They focus on comprehensive in vitro and in vivo research of the ready-made printed structures. Therefore, we undertook complex research starting from filament formation to characterization, the assessment of filament stability and the FFF 3DP process, as well as preliminary in vitro evaluation of the obtained porous structures as tissue scaffolds.

Forming of starting materials for FDM/FFF 3D printing (filaments) is a complex process which is based on the melt extrusion of polymer in the form of granules/pellets. In the literature, the process is briefly described and uses micro-scale lines, which are based on extruders equipped with a nozzle of the appropriate diameter and a manual system of diameter control [9,10,11]. There are few reports that extensively describe the processing line and parameters of the continuous filament forming process. Carneiro et al. [12] noticed that in order to obtain a dimensionally stable and defect-free filament, attention should be paid not only to the extrusion processing window but also to the appropriate equipment of the production line. Hence, the filament-forming system should also be equipped with a pulling unit, cooling/heating reservoirs, calibration zones, direct diameter measurement system or winding set. Korte at el. [13] points to the need for studies on continuous filament formation in a larger-scale production. In their research, they paid attention to selection and optimization of several extrusion parameters (pressure on the extruder head, feed rate or extrusion temperature), but also the production line itself (pulling velocity on a conveyor belt, direct laser diameter measurements, calibration zones). Thus, with the results of a full factor design of experiments (DoE), they received a pharmaceutical-grade FFF 3D printable filament in a continuous melt extrusion process. The filament-forming process is particularly challenging when the used polymers are highly flexible and possess hygroscopic nature, such as polyurethanes. Therefore, special attention should be paid to the drying process, the selection of the winding parameters and the type of cooling reservoirs, to minimize the defects of extrudate (foaming, voids, irregular diameter or generation of internal stresses).

Conducting tests, from filament formation through product application tests, ensures full control of the process and precise characteristics of the medical device printed with the FFF 3D method. Hence, in this study, polyester-urethane filament (TPU_F) was fabricated using a complex filament-forming system and the structure (FTIR, Raman, H-NMR), thermal stability (DSC, TGA), rheological (MFR) and mechanical properties, characterized. We also captured and presented line and processing parameters leading to the manufacture of high-quality TPU filament in a continuous extrusion process. Further, to find out the impact of FFF 3D printing processing on TPU_F stability, researches were carried out for both the filament and the printout (TPU_P). Then, series of in vitro studies including the evaluation of TPU_P degradability (long-term degradation in 0.1M PBS, up to six months), biocompatibility (cytotoxicity test ISO 10993:5), and determination of their surface properties (contact angle) were performed. Once the in vitro results confirmed the potential application of printouts as tissue-engineered constructs, the incubation of specially designed porous tissue structures (TPS) in simulated body fluid (SBF; 37 °C/28 days) was performed. The effect of incubation on morphology, stability, and mechanical properties of TPSs was investigated by SEM, EDS, mass measurements, and compression test. Finally, the mass loss measurements and microscopy observation during the short-term degradation (5 M NaOH; 37 °C/28 days) were also conducted. Thus, the presented paper comprehensively describes the cycle from filament production to the FFF 3DP of porous structures with various architectures and their in vitro evaluation as potential tissue scaffolds, thereby providing technological and process aspects as well as physico- chemical tests of filament stability under 3D printing conditions.

## 2. Materials and Methods

### 2.1. Materials

Epaline^®^ (390 A series) granules were purchased from Epaflex Polyurethanes (Italy). It is an extrusion grade thermoplastic polyester urethane (TPU) with a hardness of 90 Shore A, a density of 1.20 g cm^−3^, a tensile strength of 44 MPa, and elongation at break equal to 514%. Melt flow rate (MFR) is assessed as 24 g 10 min^−1^ at 5 kg load and 205 °C.

### 2.2. Filament (TPU_F) Formation

The filament-forming system was based on the melt extrusion process. Single-screw extruder with the following parameters was used, L/D ratio of 32; working length 33 mm; three-barrel heating zones; two heads heating zones; nozzle diameter of 2 mm. Several processing parameters were tested to obtain TPU_F filament with a stable diameter dimension. The scheme of the filament-forming system line with a precise description and the processing parameters is presented in Section 3.1.

### 2.3. 3D printer, Test Sample, and Porous Tissue Structure Design and Formation

Flash Forge Inventor I^®^ (FlashForge, Jinhua, China) FFF-based 3D printer with FlashPrint slicer (4.2.0 version) (FlashForge, Jinhua, China) was used to prepare testing samples. Formed TPU_F filament was used as a feedstock. Samples for the tensile test were made according to the ISO 37:2017 standard by using Autodesk Inventor software (Autodesk, Warszawa, Poland) (Table S1). Two different infill raster angles were given (0/90° and ±45°). In turn, samples for long-term degradation and cytotoxicity studies were cut out from the printed porous matrix–mesh with triangular infill of 85% and thicknesses of 3 mm (Table S2), by using brass corkscrew (Ø 8 mm). The porous mesh design was made directly in slicer software. Specimens for tensile test, long-term degradation in phosphate-buffered saline (PBS) and cytotoxicity studies (marked as TPU_P) were printed maintaining the parameters given in Table S3. Finally, three types of porous tissue structures (PTS), marked as LR, G25, and G3D and dimensions of 15 × 15 × 15 mm^3^, were designed in Autodesk Inventor software and printed with parameters given in Table S4. To increase the resolution and accuracy of printing porous structures, a 3D printer with nozzle 0.2 mm in diameter was used (Prusa MK3S, Josef Prusa, Prague, Czech Republic). PTSs differ in architecture, shape, and dimensions of the pores (Table 1). The porosity of the obtained PTSs was calculated using the following Formula (1):

(1) $$P\;\left(\%\right)=\left.\left(1-\frac{m}{V\times g}\right.\right)\times 100$$

where (*P*%) represents porosity, *m*—sample mass (*g*), *V*—the volume of the structure (cm^3^), g—density of the filament TPU_F, *g* = 1.12 g cm^−1^ (*n* = 5). 3D print accuracy was estimated according to actual dimensions of the printed PTSs (*n* = 5). Percentage values, presented in the supplementary data (Table S5), were calculated with respect to the 3D model (15 × 15 × 15 mm^3^—represents 100% accuracy).

### 2.4. Characterization Methods of Filament (TPU_F) and 3D Printouts (TPU_P)

Series of the following studies, spectroscopic (FTIR, Raman); thermal studies (DSC, TGA); gel permeation chromatography (GPC), and melt flow rate (MFR) measurements were conducted on both, filament (TPU_F) and printout (TPU_P) to characterize the obtained filament and to assess whether the printing process causes changes in the printout properties compared to the used filament.

#### 2.4.1. Spectroscopic Studies

Attenuated total reflectance (ATR) FTIR Nicolet 8700 spectrometer (Thermo Fisher Scientific, Waltham, MA, USA) was used to examine the chemical functional groups of the obtained TPU samples. The spectral range was 4000–500 cm^−1^ with 4 cm^−1^ resolution (64 scans). Measurements were taken at room temperature. In turn, Raman spectra were collected by a confocal micro-Raman system (InVia, Renishaw, New Mills, UK). Green laser (514 nm) operating at 50% of its total power (50 mW) was used. Measurements were taken at randomly selected locations on the sample surface (50× magnification).

#### 2.4.2. Thermal Properties

Thermal behavior and stability of filament and printout were studied by differential scanning calorimetry (DSC) and thermogravimetry (TG). Netzsch 204F1 Phoenix apparatus (Netzsch, Selb, Germany) was used for DSC measurements (5 mg of sample, under nitrogen atmosphere). First, the sample was heated to 220 °C, then cooled to −80 °C and finally reheated to 220 °C. The heating/cooling rate was 5 °C min^−1^. Thermogravimetric analysis was performed using a Netzsch TG 209 instrument (Netzsch, Selb, Germany) at a temperature range from 35 °C to 700 °C under nitrogen atmosphere (sample weight ~5 mg). The heating rate was 10 °C min^−1^.

#### 2.4.3. Dynamic Mechanical Analysis (DMA)

DMA Q800 analyzer (TA Instruments, New Castle, DE, USA) was used to perform dynamical mechanical tests. The measurements were carried out in the single cantilever bending mode with 1 Hz frequency of an oscillatory deformation on printed TPU samples (40 × 10 × 2 mm^3^ dimension), at the temperature range of −100 °C to 150 °C (heating rate 4 °C min^−1^). The storage modulus (G’), loss modulus (G’’), and damping factor (tangent δ) were determined as a function of temperature. 

#### 2.4.4. Melt Flow Rate (MFR)

The melt flow rate (MFR) and melt volume rate (MVR) of the obtained TPU filament and printout were measured using load plastometer (Zwick/Roell, Ulm, Germany) according to ISO 1133 standard at a temperature of 200 °C and 210 °C with a load of 5 kg. Five repetitions were performed for each sample and the results were averaged (*n* = 5).

#### 2.4.5. Hardness and Tensile Strength

The hardness measurements and tensile test were performed on printed dumbbell shaped TPU specimens. The mechanical properties of TPU_P were evaluated depending on the infill raster orientation (0/90° and ±45°). A Shore A type durometer (Zwick/Roell, Ulm Germany) was used to measure the hardness of samples (ISO 868 standard). Fifteen measurements per sample were made and the results averaged. The tensile test was conducted using a Zwick/Roell Z020 universal tensile machine (Zwick/Roell, Ulm, Germany), according to the ISO 527 standard. The study was carried out at room temperature. The crosshead speed was 100 mm min^−1^ and the initial force was 1 N. At least eight of the printed dumbbell shaped TPU series samples were tested and results were averaged (*n* = 8).

#### 2.4.6. Contact Angle (CA)

The contact angle and surface free energy (SFE) of TPU printouts were determined using a ramé-hart 90-U3 goniometer with a DROPimage Pro software (ramé-hart, Succasunna, NJ, USA). The printed surface was degreased, then 2 µL droplet of selected solvent was deposited and the images were collected. Measurements were taken over two different solvents—water and diiodomethane. The Fowkes method [14] was used to calculate SFE (based on CA results for polar (water) and non-polar (diiodomethane) liquids).

#### 2.4.7. Long-Term Degradation

The TPU printed porous meshes were subjected to long-term degradation studies to evaluate their susceptibility to degradation. Incubation was carried out in 0.1 M phosphate-buffered saline (PBS, Sigma-Aldrich) for 6 months at 37 °C. The medium was replenished every month. Porous discs with a diameter of 8 mm and a thickness of 3 mm were dried, weighed (m_0_), placed in 6 mL wells test plates, and immersed with 3.5 mL of PBS solution. At each respective time points, samples were carefully removed, rinsed out with DI water and dried in a laboratory oven at 40 °C for at least 48 h. Mass loss (Ms) was calculated as follows (2):

(2) $$Ms\left(\%\right)=\frac{m_{0}-m_{1}}{m_{0}}\times 100\%,$$

where (m_0_) is an initial mass of the sample and (m_1_) is residual mass. In the case of a mass increase due to incubation in 0.1 M PBS, the mass change (Mc) was calculated based on the following formula (3):

(3) $$Mc\left(\%\right)=\frac{m_{1}-m_{0}}{m_{1}}\times 100\%,$$

Incubation in PBS was monitored by FTIR spectra, SEM images (SEM, FEI Quanta FEG 250, accelerating voltage 10 kV), optical microscope photos of the sample surface (Delta Optical Generic Pro, Mińsk Mazowiecki, Poland), and mass measurements. Three samples were tested and the results were averaged (*n* = 3).

#### 2.4.8. Cytotoxicity Studies

Cytotoxicity of TPU printouts was studied based on ISO 10993-5 standard using fibroblast CCL-136 cell line, which was provided by ATCC, Manassas, VA, USA (BALB/3T3 clone A31, ATCC^®^ CCL-163™). Before the test, samples were sterilized under a UV lamp (Binovo, Legnica, Poland) for 30 min. Sample extracts (four different concentrations, i.e., 100, 50, 25, and 12.5%, respectively) were prepared in culture medium DMEM/F-12 with fetal bovine serum (FBS) and 5 µg mL^−1^ of penicillin with streptomycin with 5 µg mL^−1^ amphotericin B(all Corning). Solution was placed in an incubator for 24 h at 37 °C and 5% CO_2_. Next, CCL-163 cells were prepared by seeding on 96-well plates (1000 cells/well, Nunc) and incubated for 24 h in the previously prepared extract. Then, the MTT assay was conducted. The absorbance of extracts was examined via Varioskan at λ = 570 nm (ThermoFisher Scientific, Waltham, MA, USA). The statistical differences were calculated via a one-way ANOVA (α = 0.05) test (OriginPro 8.5, Washington, DC, USA).

### 2.5. Studies of Elastic Porous Tissue Structures (PTS)

In the second part of the work, printed PTSs were incubated for 28 days in simulated body fluid (SBF) solution to estimate their potential in cartilage/bone tissue engineering. Bioactivity was assessed by examination of the hydroxyapatite (HAp) crystal formulation on the sample surface via scanning electron microscopy (SEM, FEI Quanta FEG 250, accelerating voltage 10 kV, FEI, Eindhoven, Netherlands and EDS (EDAX, Mahwah, NJ, USA) (Energy Dispersive Spectrometer) spectroscopy. Further, the impact of the incubation in SBF on the mechanical properties (static uniaxial compression test, initial force 1 N, the compression rate of 20 mm min^−1^ up to 50% of the initial height, Zwick/Roell Z020, Ulm, Germany) and water contact angle of the PTS were studied. Printed PTSs were also tested in strong alkaline media. Short-term degradation was conducted for 28 days at (37 °C) in 5 M NaOH. The medium was not changed during incubation, (*n* = 5).

## 3. Results and Discussion

### 3.1. Filament (TPU_F) Formation

**Figure 1:**
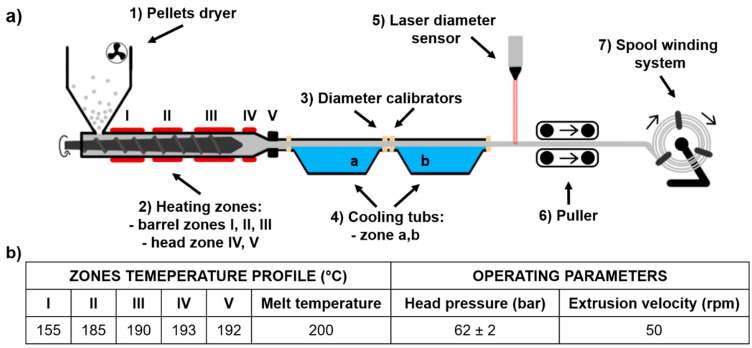
Scheme of thermoplastic polyurethane TPU filament (TPU_F) forming system (**a**), extrusion parameters of TPU_F formation (**b**).

The scheme of the used filament forming system is shown in Figure 1a. The system consisted of a single-screw extruder equipped with a granulate dryer; calibration zones with four diameter calibrators and two cooling tubs; a diameter laser sensor and the system of pulling and spool winding.

Extrusion parameters of TPU_F are shown in Figure 1b; adjusted filament forming system parameters were as follow:

(1) Pellets drying −10 h, 60 °C
(2) Hot melt extrusion parameters—Figure 1b
(3) Calibration zone (a)
  Water temperature −40 °C
  Calibrators diameter −2 mm
  Tube length ~ 2.5 m
(4) Calibration zone (b)
  Water temperature −23 °C
  Calibrators diameter −1.8 mm
  Tube length ~ 2.0 m
(5) Laser sensor accuracy −0.01 mm
(6) Pulling velocity ~ 180 rpm

**Figure 2:**
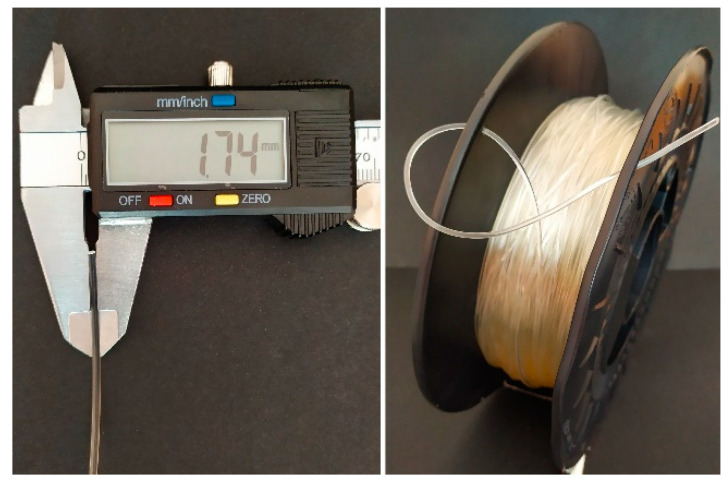
View of obtained TPU_F filament.

First, polymer pellets were dried for 10 h at 60 °C (with airflow). Then, based on the melt flow rate (MFR) value (MFR of Epaline pellets is 24 g 10 min^−1^) the temperatures and velocity of extrusion were adapted. With the set parameters, the melt temperature was 200 °C and the head pressure was kept at a constant level of 62 bar. Cooling bathtubs were refilled with water, which gradually cooled down extrudate, (40 °C and 23 °C), thus preventing the generation of thermal stress of the extrudate. This, in combination with the diameter calibrators (located at the inlet and outlet of the bathtubs), ensures that the filament is of the correct dimension and shape. The pulling velocity was adjusted in the final stage of the filament forming process (during the regulation of collecting and winding onto the spool) and was equal to 180 rpm. The mechanism controlling the diameter of a filament is most often a laser sensor or manual measurement. Herein, the filament diameter was constantly measured by a laser sensor with an accuracy of 0.01 mm. The combination of described and presented parameters resulted in the formation of polyester urethane (TPU_F) defect-free filament with a constant diameter of (1.75 ± 0.01 mm), suitable for the use in the FDM/FFF 3D printers (Figure 2).

The first step defining the success of the filament forming process is the correct preparation of the granulate (feedstock) by thorough drying. This is particularly important in the case of hygroscopic polymers such as thermoplastic polyurethanes. If water or moisture is present during extrusion, process instability may occur resulting in pressure fluctuation. This leads to the dimensional instability of the extrudate, defects in the filament (voids, bubbles), or even degradation of polymer chains. This may be manifested by a reduction in viscosity and molecular weight of the resulting filament. Hence, drying of TPU pellets is especially important. Another step of the filament forming process is adjusting the temperatures and operating parameters of melt extruding. The selection of an adequate temperature profile ensures proper plasticization of the polymer as well as affecting the head pressure in the extruder which determines the stable flow of the melt [15]. This is crucial when a specified stable diameter (filament Ø 1.75 mm) of an extruded profile is necessary. A further essential element of the process is the calibration zone. As we noted when molding highly flexible thermoplastics, it should consist of water-filled bathtubs with gradually lowering temperature. In this way, the stretching effect of the elastic extrudate, which occurs when using conventional conveyor belts, is limited. This effect disrupts the diameter stability but can also lead to buckling of the spool with the finished filament. Therefore, an incorrectly selected calibration and the winding system can lead to permanent damage to the spool with the highly flexible filament (see Figure S1).

### 3.2. Chemical Analysis of TPU Filament and Printout

**Figure 3:**
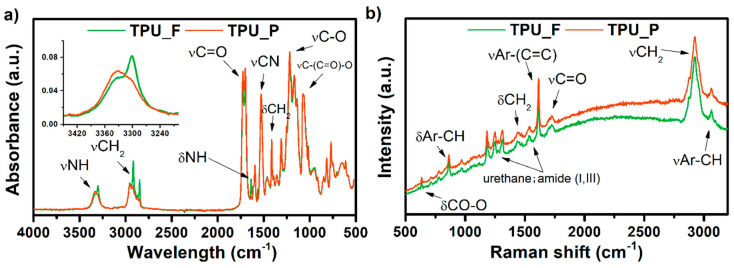
FTIR (**a**) and Raman (**b**) spectra of obtained TPU filament and printout.

The results of the spectroscopic studies of formed TPU_F and its printout (TPU_P) are shown in Figure 3. The presence of functional groups characteristic for polyester urethanes was noted, based on the FTIR spectrum (Figure 3a). The absorption peak visible in the range of 3300 cm^−1^ corresponds to –NH group stretching vibrations of the urethane blocks. Bending vibration of –NH and stretching vibration of C–N of urethane blocks are seen at 1594 and 1525 cm^−1^, respectively. The range of 2950 cm^−1^ is associated with symmetrical and asymmetrical stretching vibration of aliphatic –CH_2_ groups. The signal observed at 1750 cm^−1^ is associated with C=O stretching vibration (both, hydrogen and non-hydrogen bonded) in polyester and urethane groups. Peaks noticed between 1170–1070 cm^−1^ correspond to vibration of urethane units; stretching vibration of C–O and C–(C=O)–O.

In the case of the TPU_F sample, two bands appear in this region. The peak at 3330 cm^−1^ corresponds to free –NH groups while at 3300 cm^−1^ the –NH groups are hydrogen-bonded [16]. After the FDM 3D printing process (TPU_P), the band slightly shifts towards higher values, and the peak representing the hydrogen-bonded –NH group decreases significantly. Thus, the printing process reduced the number of hydrogen-bonded –NH groups occurring in the TPU structure. Nevertheless, material degradation due to the 3D printing process cannot be stated.

The Raman spectrum confirmed the structure of the obtained TPU filament. Amide bands (I, III) were detected. The presence of peaks corresponding to the vibrations of aromatic carbons was also observed (stretching vibration of aromatic CH at 3064 cm^−1^ and C=C of benzene ring at 1620 cm^−1^) which is most likely derived from the aromatic diisocyanate used for polyester urethane synthesis. The above considerations are consistent with the ^1^H-NMR measurements which showed the presence of methylene diphenyl diisocyanate (MDI) in the TPU_F structure (see supplementary data, Figure S2).

### 3.3. Thermal and Thermomechanical Properties of TPU Filament and Printout

**Figure 4:**
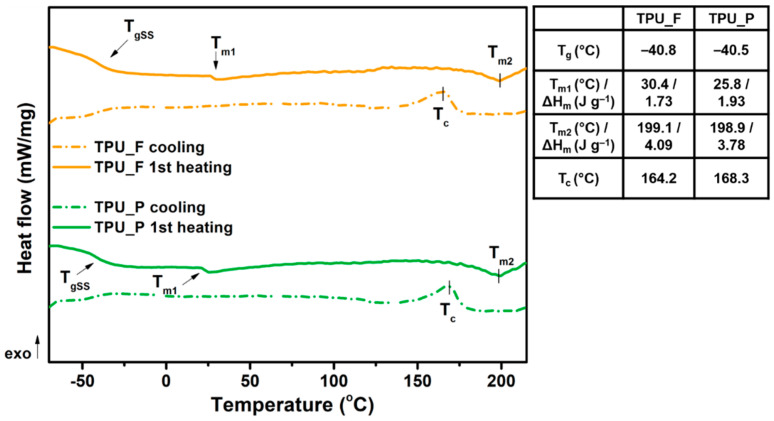
Differential scanning calorimetry DSC curves of obtained TPU filament and printout (1st heating and cooling cycle).

The obtained filament (TPU_F) and printout (TPU_P) samples were subjected to a series of thermal and thermo-mechanical tests. Thermoplastic polyurethanes built with hard and soft segments show the ability for micro-phase separation and formulation of the microstructure [17]. Crystallizable phases of TPU microstructures can be studied by differential scanning calorimetry (DSC) measurements. DSC thermograms (Figure 4) enabled the determination of the glass transition temperature (T_g_), melting temperature (T_m_), heat enthalpy (ΔH_m_), and crystallization temperature (T_c_). The first heating scans of both samples reveal T_g_ to be in a very similar range of ~40 °C. Therefore, it can be assumed that the 3DP process did not affect the soft phase transition of TPU. Two melting points were observed. T_m1_ corresponds to the endothermic transition of soft segments, while T_m2_ is connected with the melting of crystalline hard segments present in the TPU structure. The heat enthalpy value of both transitions is relatively low, which suggests a low crystallinity degree of the studied samples. It is worth noting that the T_m1_ of TPU_P was about 5 °C lower than TPU_F (25.8 °C) whilst the melting temperature of hard segments did not change. The crystallization temperature of hard segments was noticed between 164–168 °C. A possible cause of the T_m1_ shift may be a change in the mobility of the soft segment chains due to the 3DP process. Nevertheless, the variations are inconsiderable and do not indicate TPU_P degradation.

**Figure 5:**
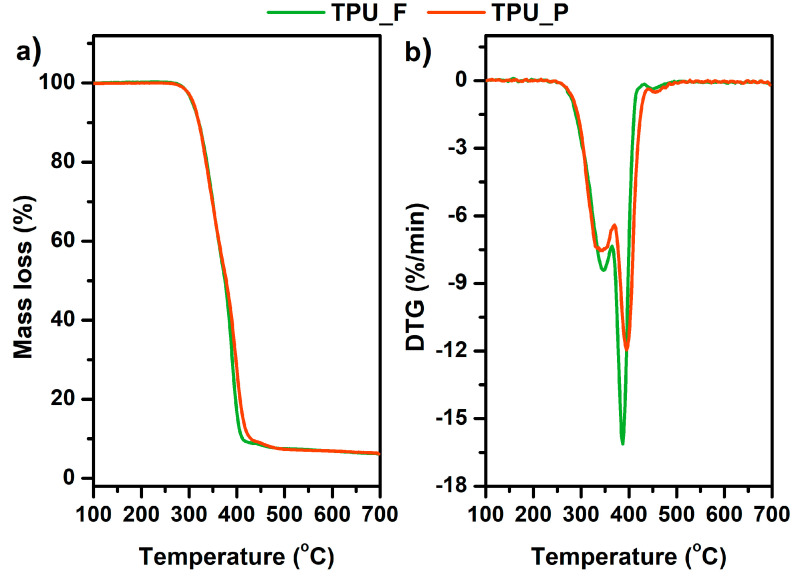
Mass loss (**a**) and derivative thermogravimetric DTG curves (**b**) vs. temperature of TPU filament and printout.

Thermogravimetric analysis (TGA) allowed the determination of the thermal stability of the prepared samples. The results are presented in Table 2 and Figure 5. The thermal stability of both samples was similar, around 300 °C (T_onset_). The complete decomposition of material occurred at 475 °C (T_offset_). Since thermoplastic polyurethanes have a segmented structure, the degradation of these polymers takes place in two stages. Additional information on the phase separation of TPU can be provided by the first derivative of the TGA curve (DTG) diagrams in which clearly separated peaks indicate the decomposition of subsequent polyurethane phases. The determination of DTG curves indicates a two-stage material decomposition, which confirms the segmental structure of the tested polyurethanes [18]. The first peak on the DTG curves corresponds to the decomposition of the urethane bonds that build hard segments (at ~350 °C), while the destruction of the soft segments (ester bonds), present in long-chain polyester-part of TPUs, begins at 390 °C. Such minor changes in thermal properties between TPU filament and printout allow it to be stated that the formed filament is thermally stable under FFF 3D printing conditions.

For further characterization of the obtained material, dynamic mechanical analysis (DMA) was performed. DMA analysis is used to determine the mechanical and viscoelastic properties of materials as a function of temperature, time, and frequency.

**Figure 6:**
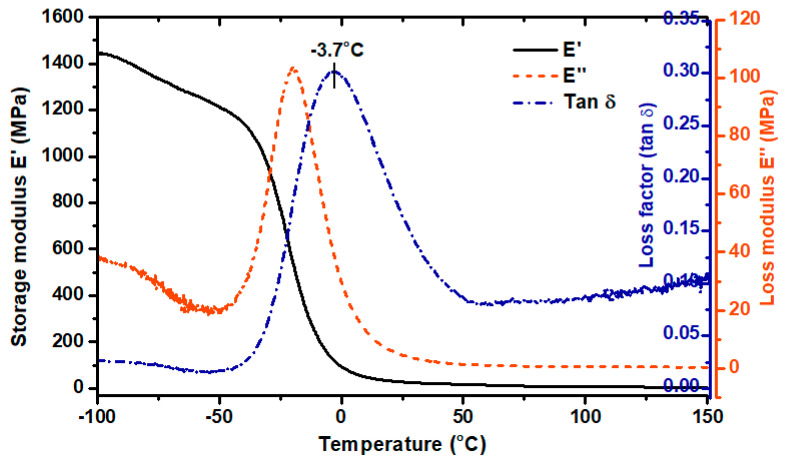
Dynamic mechanical analysis DMA results of TPU printout. Storage modulus (E’), loss modulus (E’’), loss factor (Tan δ).

Based on the curves shown in Figure 6, different viscoelastic states of the sample can be distinguished, i.e., at minus temperature range—glassy state; a broad glass transition region (−40 to 40 °C); a rubbery plateau region (over 50 °C). The value of the storage modulus (E’) in the glassy state region was over 1450 MPa and decreased by about 1.2 times after exceeding −40 °C. The α-relaxation temperatures, determined as the values of the glass transition region start, a maximum value of E’’, and loss factor (tag δ) peak, were −39 °C, −21 °C and −3.7 °C, respectively. In this temperature range, the long-rate intermolecular movements of the macromolecule polymer chains take place. The maximum amplitude of loss factor (so-called damping factor) reached 0.3 which indicates scanty damping properties of the formed sample. According to the literature, polyurethanes designed to dampen vibration (with excellent energy dissipation) exhibit a loss factor value (tag δ) greater than 0.6 [19,20].

### 3.4. Melt Flow Rate (MFR) Results

The melt flow is a parameter determining the polymer’s ability to melt and flow under the influence of temperature and pressure. It can be expressed in units of mass (MFR) or volume (MVR) of the material flowing from the plastometer nozzle under a given load over a period of 10 min. The conditions during the MFR measurement can be compared with those in the print head during FFF 3D printing. MFR is also related to the dynamic viscosity of the polymer melt, so it can be useful in assessing the printing speed at which the filament can be successfully printed. The studies conducted by Ramanath et al. [21] showed that MFR is closely related to the interlayer adhesion between formed layers and therefore affects the quality of the printed part. Therefore, the melt flow rate is a very helpful parameter in evaluating the suitability of a filament for FFF 3DP [22].

The results of the melt flow measurements are listed in Table 3. The study showed an increase in the MFR value after the 3DP process. Hence, the rheological properties of the formed TPU filament changed slightly. This is related to the reprocessing of the material during 3D printing in which changes in the polyurethane microstructure occur. It is in line with the thermal characteristics as well as with results of the FTIR examination, wherein a small reduction in the number of hydrogen bonds, through which the domain structure in polyurethanes is formed, was noticed. However, taking into account the standard deviation of the results, this difference is insignificant.

The MFR measurement for the TPU_F sample proceeded smoothly. The material-flow from the plastometer nozzle was continuous, without any swelling effect. No defects of extrudate (bubbles, foaming) were observed. The MFR value of the TPU_F was around 22 g 10 min^−1^ at 200 °C and a load of 5 kg and increased by ~8 g 10 min^−1^ with increasing test temperature by 10 °C. The melt flow of commercial TPU filaments is around 16 g 10 min^−1^ (Ultimaker^®^, TPU 95A, 225 °C/1.2 kg), 15 cm^3^ 10 min^−1^ (Basf^®^, Ultrafuse TPU 85A, 190°C/3.8 kg). However, it is difficult to compare these values because they strictly depend on the measurement conditions which were variable in each case.

During the stage of adjusting the 3D printing parameters of TPU_F, it was noticed that the printing temperature of 210 °C ensures the correct course of the process with the best ratio of time to printout quality. Hence, it can be concluded that the MFR values of the order of ~20–30 g 10min^−1^ (at a temperature range of 200–210 °C and 5 kg load) allow to pre-qualify the studied TPU filaments as suitable for 3D printing with the FFF/FDM method. However, it should be remembered that to fully assess the suitability of the filament for 3D printing, the results of tests such as thermal, rheological or mechanical should be taken into account [23,24,25].

### 3.5. Hardness and Tensile Test of TPU_P

Further analysis concerns only the printed samples (TPU_P) to demonstrate the practical application of the formed filament (TPU_F). Series of conducted mechanical studies have shown that the change in printing parameters (infill orientation) did not significantly affect the hardness and tensile strength profile of the samples (Table 4). Although the mean values for the samples marked as ± 45° are higher, the standard deviation indicates that the mechanical properties are not strictly related to the infill orientation. It allows the assumption that the printouts showed good interlayer adhesion, and therefore the orientation of the sequentially arranged/placed fibers did not affect the obtained results.

The hardness of printed structures was in the range of 85 ShA (~33 ShD) which corresponds to a type of hard rubber. Tested samples showed a comparatively low value of the relative elongation parameter (~30–40%), which indicates a small permanent deformation formed after loading the printouts. It is worth paying attention to the decrease in mechanical properties in relation to the raw-material (Epaline^®^ granules) from which the filament was formed. The tensile strength, elongation at break and hardness of Epaline^®^ was 44 MPa, 514%, and 90 ShA, respectively. This corresponds to a decrease in mechanical properties of 30, 20, and 5% concerning the starting material. It is related to the imperfections of FFF 3D printing technology in which defects in the print structure, such as voids, warping deformations or insufficient interlayer bonding (i.e. raster-raster, contour raster bonding) caused by uneven temperature distribution, cannot be overcome [26,27,28].

### 3.6. Results of in vitro Studies on TPU_P

#### 3.6.1. Long-Term Degradation in PBS

**Figure 7:**
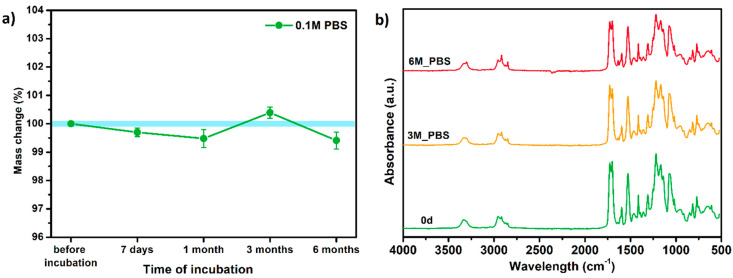
The results of long-term degradation studies on TPU_P samples in 0.1 M phosphate-buffered saline PBS solution (37 °C), (**a**) mass change measurements, (**b**) FTIR spectra. Optical images can be found in supplementary data Table S6.

Since TPU filament was to be considered as a material for medical applications a series of in vitro studies was performed. Initially, long-term degradation in the phosphate-buffered saline (PBS) environment, to verify the stability of the material under simulated conditions, was performed [29,30]. The long-term degradation was monitored by mass measurements, optical microscopy, SEM images, and FTIR study (Figure 7, Table 5 and Table S6).

The mass measurements showed that the TPU_P samples are stable for up to six months of incubation in PBS. Weight changes did not exceed 1% throughout the study. The stability of the printouts was confirmed by FTIR study. The spectrum of the sample remained unchanged after six months of incubation. SEM images showed that the morphology of TPU_P did not change during incubation, no surface erosion or cracking was observed. At the end of the study, however, yellowing of the sample was noticed (Table S6), most likely due to accumulation on the sample surface of salt deposits. Nevertheless, the stability of TPU_P, under PBS incubation conditions, corresponds to the requirements set for structures intended, for instance for tissue engineering application [31].

#### 3.6.2. Biocompatibility and Surface Properties

Further in vitro studies included the evaluation of printout biocompatibility and examination of their surface properties. One of the basic tests evaluating the suitability of materials intended for medical applications is the cytotoxicity test [32]. It uses tissue cells in vitro and monitors their response to contact with foreign material (cell growth, viability, and morphological effect).

**Figure 8:**
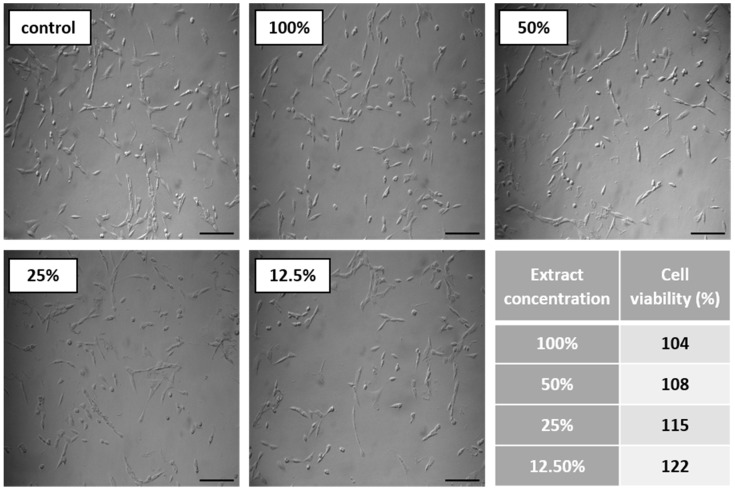
Results of cytotoxicity test. Images show CCL-163 cells morphology after 24 h contact with TPU_P extracts of specified concentrations (100–12.5%). Scale bar = 50 µm. The table present percentage of cell viability towards control (100%). SD were negligibly small, there were no statistical differences between the obtained results.

The cytotoxicity test results are displayed in Figure 8. Noted cell viability after 24 h of exposure against all prepared TPU_P extract was greater than 100%, indicating cell proliferation. The prepared TPU_P extracts did not show a toxic effect against the fibroblast cells. The images of CCL-163 cells after 24 h contact with TPU_P extracts—show no morphological changes in relation to the control. Consequently, the TPU_P can be pre-defined as biocompatible and non-toxic [33]. The obtained results are in common with the in vitro cytotoxicity studies on polyurethanes materials, performed by Calvo-Correas et al. or Uscátegui et al. [34,35]. Thus, the statement, common in the literature, that polyurethanes are widely used in medical applications due to their exceptional biocompatibility and non-toxicity was confirmed [36].

The biocompatibility and thus the adhesion, proliferation, and differentiation of cells, are reflected in the surface properties of a biomaterial [37]. The surface properties, among others, can be defined by measuring the contact angle (CA) and surface free energy (SFE). The generally accepted definition says that hydrophilic materials exhibit a water contact angle of less than 90°, while hydrophobic materials have CA>90° [38]. Medical-grade polyurethanes show a wide range of CA depending on the designed chemical structure and desired application. For example, the polyurethane microporous thin layers (MPTL) intended for soft tissue engineering, obtained by Kucinska-Lipka et al., possessed CA of 55° [39], whereas electrospun PUR bone scaffolds exhibited strongly hydrophobic properties (CA over 100°) [40]. Therefore, when considering the use of the obtained samples in tissue engineering, it should be taken into account that an excessively hydrophilic surface can disrupt the interaction between seeded cells, while too high hydrophobicity may lead to a reduction in biocompatibility due to hindered cells’ adhesion [37].

The water contact angle of TPU_P was of 76° while SFE was of 42 mN m^−1^, which indicates a quite hydrophilic nature with moderate wettability (Table 6). Taking into account the results of performed in vitro tests on TPU_P samples, it can be stated that the studied printouts meet the initial requirements for materials distorted for tissue engineering, i.e., they are stable for up to six months in PBS incubation conditions; they are non-toxic towards CCL-163 cells line, and show promising surface properties. Therefore, in the next step, specially designed porous structures were printed using TPU_F and tested to determine their potential as tissue scaffolds.

### 3.7. Porous Tissue Structures (PTS) after Exposure in Simulated Body Fluid (SBF) and Their Susceptibility to Accelerated Degradation (5 M NaOH)

SBF incubation is a well-known method for the preliminary determination of the in vitro bioactivity of materials [41]. Since the SBF solution possesses ion concentration nearly equal to those of human blood plasma, the formulation of bonelike apatite on a material surface can be monitored. While the mineralization effect of bone-like implants is highly desirable [42], in the case of soft tissue structures (e.g., heart valve, coronary stents, urinary implants) the process can be disastrous [43].

**Figure 9:**
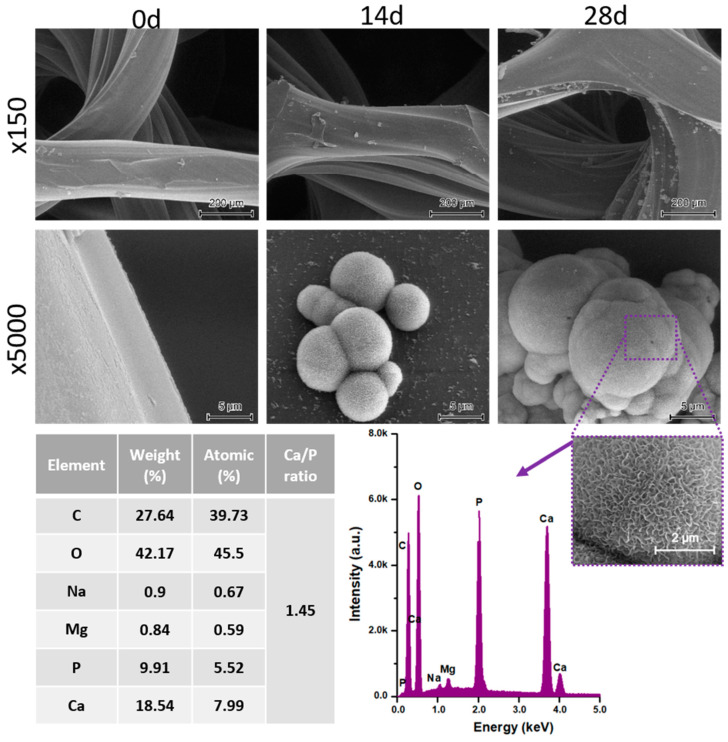
SEM images and EDS spectrum of G25 PTS after incubation in SBF.

The printed porous tissue structures (PTS) with different architecture (LR, G25, G3D, Table 1) were subjected to incubation in simulated body fluid (SBF) for 28 days. The SEM examination revealed the appearance of spherical particles on the surface of the PTS, already after 14 days of soaking in SFB solution (Figure 9). They were randomly located on the entire surface area of the studied porous structures, creating spherical agglomerates. The particle morphology resembled a micro lath-like network, with a structure typical for hydroxyapatite (HAp) minerals [44,45]. EDS study of the crystals revealed a high content of the elements Ca, P, O, and C, respectively, which correspond to the chemical formula of hydroxyapatite (Ca_10_(PO_4_)_6_(OH)_2_). The calcium to phosphate (Ca/P) atomic ratio of that phase was equal to 1.45. The Ca/P value of stoichiometric HAp was 1.67, thus the formed minerals seem to be deficient in calcium. The reason might be the presence of Mg^+^ and Na^+^ ions, which distort the structure of HAp. However, it is worth noting the relatively high content of the carbon and oxygen elements in the crystal structure, which suggest the presence of CO_3_^−^ ions. Thus, on the PTS surface, so-called carbonated-hydroxyapatite (C-HAp) was formed. According to the literature, C-HAp corresponds more to natural-occurring hydroxyapatite and therefore possesses enhanced biocompatibility compared to the synthetic one [45,46].

**Figure 10:**
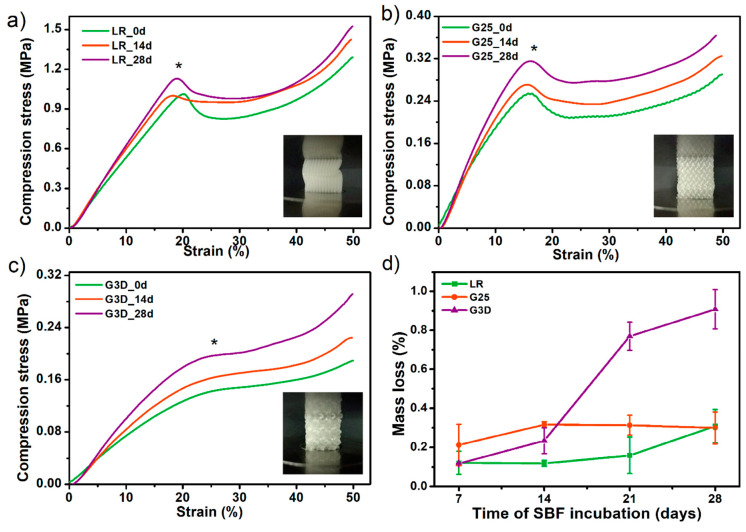
The influence of incubation in simulated body fluid SBF solution on compressive strength (**a**–**c**) and mass loss (**d**) of selected porous tissue structures PTS. * The inflexion point of the stress-strain curves (the so-called Yield point) was assumed as the compressive strength values of studied PTS.

Further, SBF-incubated PTS samples were subjected to a compression test and the results were compared with the values before incubation (Figure 10a–c). The LR-PTS structure proved to have the most compressive strength (~1.05 MPa) while the G3D type - the lowest one (~0.12 MPa). It was also observed that with the change of the porous structure architecture (porosity and pore shape/size), the course of the compression curve changes. The LR and G25 samples show a clear yield point and a sharp increase in stress, while the G3D structure (most irregular) shows a mild increase in stress over compression. Moreover, in all type of PTS, an evident increase in compressive strength with the incubation time in SBF was observed. This increase was the highest for the G3D and G25 samples, by around 0.08 MPa and 0.07 MPa, respectively after 28 days of the incubation period. Thus, the mineral particles of hydroxyapatite formed during SBF soaking significantly strengthened the elastic porous structures. The addition of hydroxyapatite (synthetic or natural) to the polymeric tissue scaffolds most likely increases their compressive strength [47,48]. However, the process of isolation HAp from the SBF solution involves simultaneous hydrolytic degradation, thus a mass loss of the sample. Therefore, in many cases, a decrease (or no change) in the mechanical properties of polymeric tissue scaffolds is observed as a result of their incubation in SBF solution [49,50]. The mass loss of printed PTS after 28 days of SBD soaking did not exceed 1% (Figure 10d), so an intense strengthening effect of the secreted C-HAp was observed. This study confirmed the previous results of degradation in 0.1 M PBS and hence the PTSs can be considered as long-term scaffolds for bone tissue engineering.

**Figure 11:**
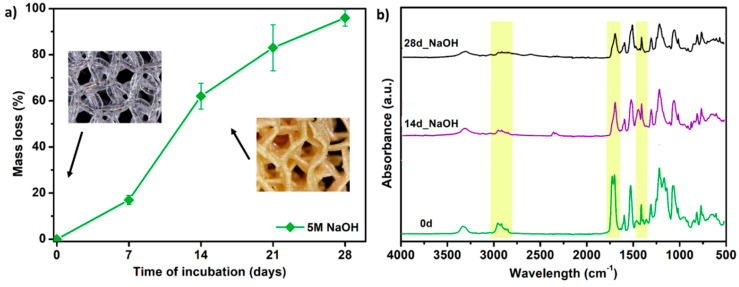
The results of accelerated degradation studies on G25 PTS in 5 M sodium hydroxide NaOH, (**a**) mass loss measurements, (**b**) FTIR spectra. SEM images showing the accelerated degradation process are presented in supplementary data (Table S7).

Finally, the selected PTS sample (G25) was subjected to accelerated degradation study in 5 M NaOH for 28 days. It is a widely used method for short-term in vitro evaluation of the degradability of medical-grade materials [51]. Together with testing in PBS and SBF solutions, this completes the material degradation profile. The results of the study in terms of mass loss and FTIR measurements are presented in Figure 11. Already after 7 days of incubation, a weight loss of the G25 sample by 18% was noted. Whereas on the 14th day of the test, the sample structure was destroyed and the material was fully degraded (mass loss ~ 60%). The FTIR spectra showed that degradation proceeds mostly by destruction of the ester (C=O 1750 cm^−1^) and urethane (–NH 1594 cm^−1^, –CN 1525 cm^−1^) bonds. Aliphatic –CH_2_ groups presented in long-chain hydrocarbon chains were also destroyed (2950, 1320 cm^−1^). Thus, the G25 PTS is very susceptible to erosion in a highly alkaline environment which is in the line with polyester polyurethanes as they are susceptible to hydrolysis in an alkaline media [52,53]. Some of the materials dedicated to tissue engineering applications exhibit mass loss around 80–90% during a month of accelerated degradation (or ~50% up to 15 days of alkaline incubation), thus the obtained PTSs might find such application as long-term degradable and elastic tissue scaffolds [51,54,55].

## 4. Conclusions

In this work, filament processing, its comprehensive characterization, and the FFF 3D printing of elastic porous tissue structures were presented and described extensively. Since FFF 3DP in medical applications is widely applied, studies of novel filament solutions are desirable. Polyurethanes, as proven materials used in the medical industry with a wide range of properties, among others are attractive in bone-tissue engineering application. Therefore, here we presented fully characterized polyester-urethane filament with potential in 3D printing of elastic porous structures for cartilage tissue engineering.

The most important conclusions of the study are as follows:

- The full list of filament-forming parameters which ensures the formation of a stable flexible filament was presented. Besides, the critical points of the process occurring during the formation of filament for FDM/FFF 3D printing were described.
- Spectroscopic studies (FTIR, Raman) confirmed the structure of the obtained TPU filament. Moreover, the analysis of spectra confirmed that the FFF 3D printing process does not cause significant structural changes in the formed filament.
- The series of thermal and thermomechanical tests showed that the TPU_F is stable under the FFF 3DP process.
- By analyzing the MFR results, the range of values that allows prediction of the suitability of the tested flexible filament for 3D printing in FFF technology was determined. The MFR range is 20–30 g 10 min^−1^ (at 200–210 °C and 5 kg load).
- A series of in vitro tests showed that TPU_P printouts are stable for up to 6 months under PBS incubation conditions, meet the cytocompatibility requirements (ISO 10993:5), and have a water contact angle of ~76°. Thus, the studied material can be considered in medical applications, including tissue engineering.
- The results of accelerated degradation point out that the formed PTSs exhibit degradability suitable for long-term tissue engineering structures.
- Incubation of PTSs in SBF solution showed the susceptibility to mineralization and formation of hydroxyapatite (HAp) crystals on their surface. This effect is desired in the case of bone-like systems. Nevertheless, to confirm the bioactivity of PTS, further, more advanced biological studies must be carried out.
- HAp released on the surface of the samples significantly strengthened the examined structures. There was up to 55% increase in compressive strength after incubation in SBF for 28 days (for the G3D sample).
- The scaffold architecture (pore shape, porosity) significantly affects the mechanical properties. The LR-type architecture with the highest degree of pore ordering exhibited the highest compressive strength of 1.1 MPa.
- The tensile strength and Young’s modulus values of the obtained printouts and compression strength of the formed PTSs, enable pre-consideration of TPU_F for FFF 3DP for application in cancellous bone tissue engineering (Table 7).

## Figures and Tables

**Table 1:** Preview of prepared porous tissue structures (PTS) (model, printout, pores pattern).

Type of PTS	LR	G25	G3D
Side view of the project	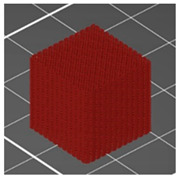	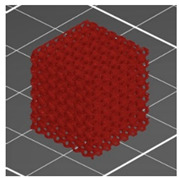	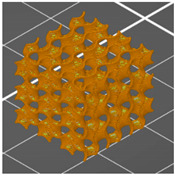
Top view of the project	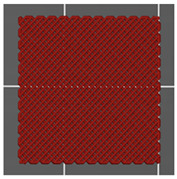	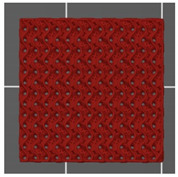	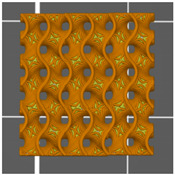
Printout	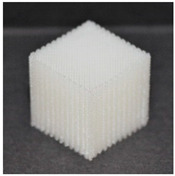	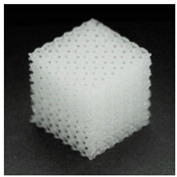	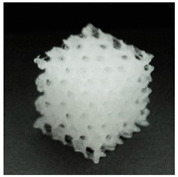
Pores pattern	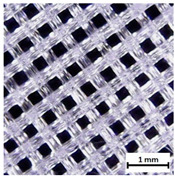	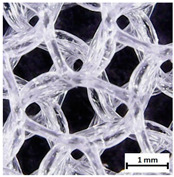	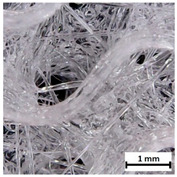
Porosity (%)	58.4 ± 1.2	76.6 ± 0.4	78.8 ± 0.6
Print accuracy (%)	x − 98.95 ± 0.18 y − 98.87 ± 0.16 z − 98.77 ± 0.15	x − 98.77 ± 0.10 y − 98.73 ± 0.16 z − 98.84 ± 0.22	x − 97.17 ± 0.33 y − 97.45 ± 0.26 z − 98.67 ± 0.08

**Table 2:** Thermal decomposition characteristics of the TPU filament and printout.

Sample	T_5%_ (°C)	T_50%_ (°C)	T_max_ (°C) Step I/Step II	Residual Mass at 700 °C (%)
TPU_F	307.3	375.1	351.8/390.3	6.15
TPU_P	308.8	377.9	347.0/394.6	6.34

**Table 3:** The results of melt flow measurements of the TPU filament and printout.

Sample	Temperature 200 °C, Load 5 kg		Temperature 210 °C, Load 5 kg	
	MFR (g 10 min^−1^)	MVR (cm^3^ 10 min^−1^)	MFR (g 10 min^−1^)	MVR (cm^3^ 10 min^−1^)
TPU_F	21.8 ± 0.4	20.3 ± 0.3	29.7 ± 0.7	27.9 ± 0.8
TPU_P	23.9 ± 1.1	22.5 ± 1.4	32.1 ± 0.9	30.3 ± 1.1

**Table 4:** Results of hardness and tensile strength test. The conditions of test sample printing are shown in the supplementary appendix.

Infill Orientation	HS (Sh A)	Tensile Strength (MPa)	Elongation at Break (%)	Relative Elongation (%)	Young’s Modulus (GPa)
± 45°	86 ± 2	31.1 ± 6.5	412.6 ± 53.4	39.1 ± 7	0.21 ± 0.10
0/90°	85 ± 1	29.0 ± 3.2	392.3 ± 42.8	32.2 ± 2	0.19 ± 0.05

**Table 5:** Scanning electron microscope SEM images of long-term degradation of TPU_P (day 0, 3 and 6 months of study).

	0 d	3 M	6 M
	**Top View**		
×100	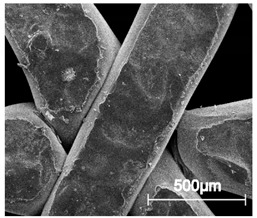	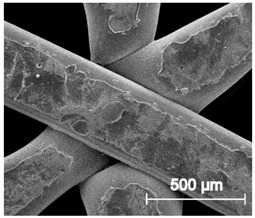	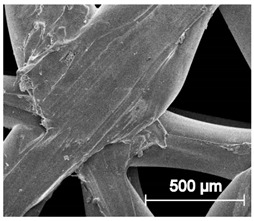
×250	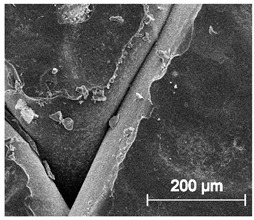	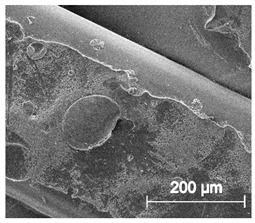	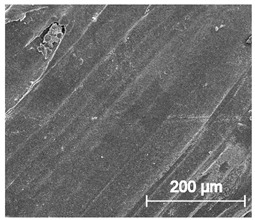
×1000	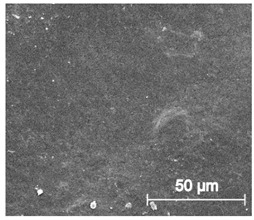	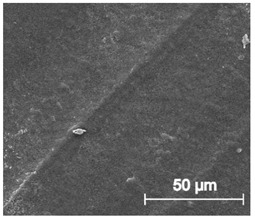	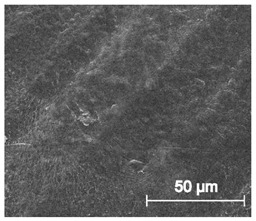
	**Cross view**		
×100	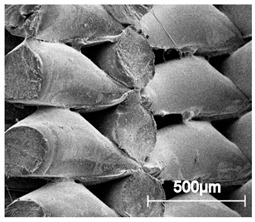	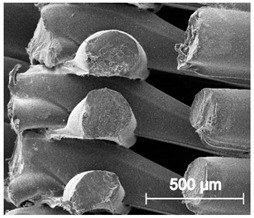	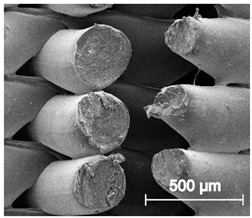
×250	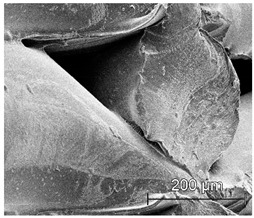	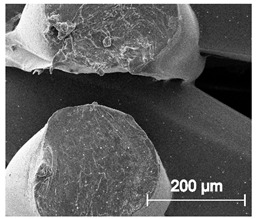	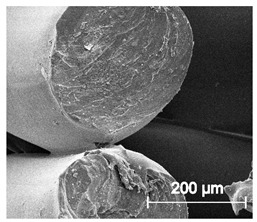
× 1000	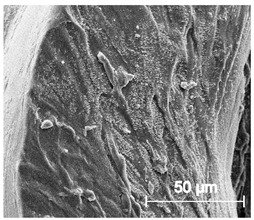	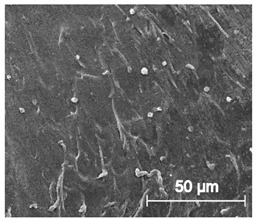	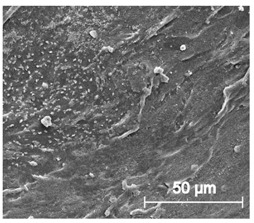

**Table 6:** Result of contact angle measurements of TPU_P.

Water Contact Angle (°)	Diiodomethane Contact Angle (°)	Surface Free Energy (mN m^−1^)		
75.9 ± 3.5	43.7 ± 2.7	Dispersive part 37.6 ± 0.2	Polar part 5.0 ± 0.1	Total 42.6 ± 0.2

**Table 7:** Summary of the properties of TPU_F printed structures vs cancellous human bone.

	Young’s Modulus (GPa)	Tensile Strength (MPa)	Compressive Strength (MPa)
**TPU_F FFF printouts**	0.19–0.21	29–31	0.16–1.20
**Cancellous boneReference**	0.1–0.5 [56]	1–5 [56]	4–12 [56] 0.1–16 [57]

## Data Availability

The raw/processed data required to reproduce these findings cannot be shared at this time due to technical or time limitations. Nevertheless, they can be made available on request.

## Supplementary Materials

The following are available online at https://www.mdpi.com/1996-1944/13/19/4457/s1, Figure S1: Damaged spool of TPU filament due to excessively high extrudate stresses generated during the forming and winding process, Figure S2: ^1^H-NMR spectra of TPU_F and TPU_P, Table S1: Design of prepared dumbbell shaped TPU samples for tensile test, Table S2: Design of porous matrix for degradation studies of TPU, Table S3: 3D printing settings of test samples TPU_P (dumbbell shaped tensile test specimens, porous matrix for long term degradation in PBS and samples for cytotoxicity study), Table S4: 3D printing settings of porous tissue structures with different architecture (LR, G25 and G3D), Table S5: Analysis of PTSs print accuracy, Table S6: Optical microscopy images of TPU_P samples obtained during long-term degradation study in 0.1 M PBS solution, Table S7: SEM images from the accelerated degradation studies of G25 porous tissue structure.
