# Supplementary material for: Processing of Polyester-Urethane Filament and Characterization of FFF 3D Printed Elastic Porous Structures with Potential in Cancellous Bone Tissue Engineering
Source: Materials (Basel). 2020 Oct 8;13(19):4457. doi: 10.3390/ma13194457 (PMC7579379; doi:10.3390/ma13194457)
Supplement: Supplementary file 1 [file materials-13-04457-s001.pdf]

materials

MDPI

Supplementary

Processing of Polyester-Urethane Filament and Characterization of FFF 3D Printed Elastic Porous Structures with Potential in Cancellous Bone Tissue Engineering

Agnieszka Haryńska *, Iga Carayon, Paulina Kosmela, Anna Brillowska-Dąbrowska *, Marcin Łapiński, Justyna Kucińska-Lipka and Helena Janik

Supplementary data

Dumbbell-shaped specimens for the tensile test (Table S1)

Table S1. Design of prepared dumbbell-shaped TPU samples for tensile test.

Specimen Dimensions	Raster Angle	Printout
➢ A =115<br>➢ B=25<br>➢ C=33<br>➢ D=6<br>➢ E=14<br>➢ F=25<br>➢ G=2<br>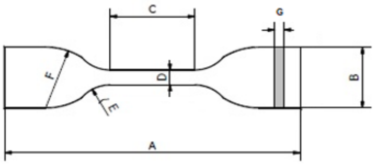	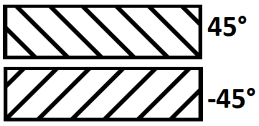	±45<br>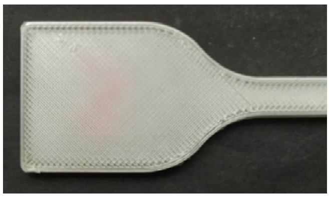
View of STL file<br>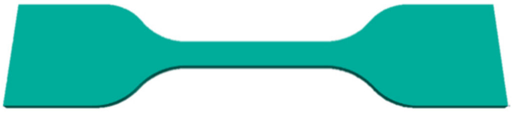	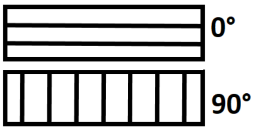	0/90<br>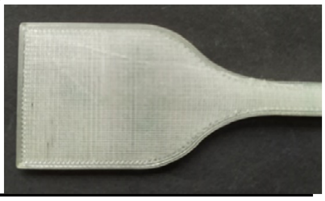

Porous matrix for long-term degradation in PBS and cytotoxicity studies (Table S2)

Table S2. Design of porous matrix for degradation studies of TPU.

View of STL file	Printout
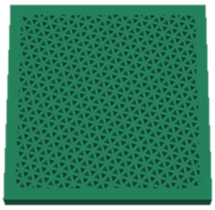	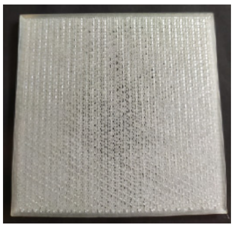
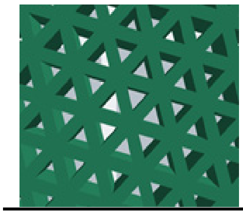	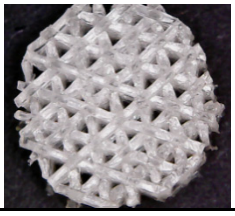

3D printing parameters (Tables S3 and S4)

Table S3. 3D printing settings of test samples TPU_P (dumbbell shaped tensile test specimens, porous matrix for long term degradation in PBS and samples for cytotoxicity study).

3D Printing Parameter	Value
Extrusion temperature (°C)	210
Bed temperature (°C)	65
Cooling	Fan on (start from the second layer)
Printing speed (mm s^{-1})	20
Layer height (mm)	0.18
Infill (%)	100
Nozzle diameter (mm)	0.4
Extrusion ratio (-)	1.05

Table S4. 3D printing settings of porous tissue structures with different architecture (LR, G25 and G3D).

3D Printing parameter	LR	G25	G3D
Extrusion temperature (°C)		225	
Bed temperature (°C)		60	
Cooling		Fan on (start from the second layer)	
Printing speed (mm s^{-1})		20	
Layer height (mm)		0.15	
Extrusion ratio (-)		1.00	
Nozzle diameter (mm)		0.2	
Print time (min)	46	31	49
Used filament (m)	0.73	0.36	0.32

3D print accuracy measurements of printed TPSs

Table S5. Analysis of PTSs print accuracy.

PTS Symbol	3D Model Dimensions (mm)	Actual Dimensions of Printouts (mm) x, y, z (Directions)	Print Accuracy (%) x, y, z (directions)	Average Value of Print Accuracy (%)
LR	15 × 15 × 15	14.82 × 14.77 × 14.80	98.80 × 98.47 × 98.67	x—98.95 ± 0.18<br>y—98.87 ± 0.16<br>z—98.77 ± 0.15
		14.81 × 14.82 × 14.79	98.73 × 98.80 × 98.60	
		14.85 × 14.84 × 14.87	99.00 × 98.93 × 99.13	
		14.90 × 14.87 × 14.82	99.33 × 99.13 × 98.80	
		14.83 × 14.85 × 14.80	98.87 × 99.00 × 98.67	
G25		14.81 × 14.85 × 14.87	98.73 × 99.00 × 99.13	x—98.77 ± 0.10<br>y—98.73 ± 0.16<br>z—98.84 ± 0.22
		14.80 × 14.78 × 14.80	98.67 × 98.53 × 98.67	
		14.80 × 14.79 × 14.77	98.67 × 98.60 × 98.47	
		14.85 × 14.80 × 14.84	99.00 × 98.67 × 98.93	
		14.82 × 14.83 × 14.85	98.80 × 98.87 × 99.00	
G3D		14.59 × 14.63 × 14.81	97.27 × 97.53 × 98.73	x—97.17 ± 0.33<br>y—97.45 ± 0.26<br>z—98.67 ± 0.08
		14.52 × 14.57 × 14.81	96.80 × 97.13 × 98.73	
		14.64 × 14.67 × 14.77	97.60 × 97.80 × 98.47	
		14.51 × 14.57 × 14.81	96.73 × 97.13 × 98.73	
		14.62 × 14.65 × 14.80	97.47 × 97.67 × 98.67	

The effect of the buckled spool.

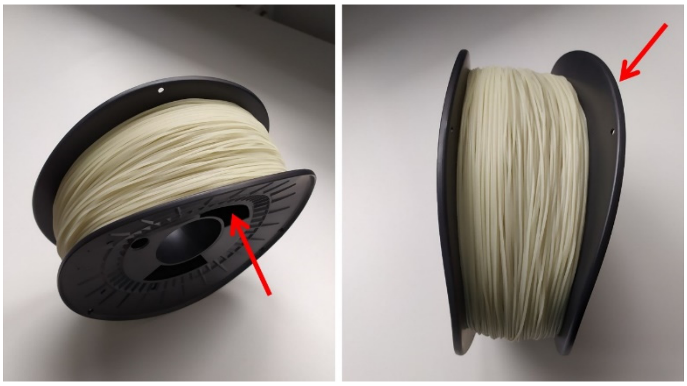

Figure S1. Damaged spool of TPU filament due to excessively high extrudate stresses generated during forming and winding process.

Nuclear magnetic resonance ^{1}H-NMR

Proton nuclear magnetic resonance (^{1}H-NMR) spectra were obtained using Bruker Avance III HD spectrometer (400 MHz). 10 mg of samples were dissolved in 1 mL of deuterated dimethylsulfoxide (DMSO-d6, Sigma-Aldrich). The TopSpin® software (Bruker) was used to process the obtained data.

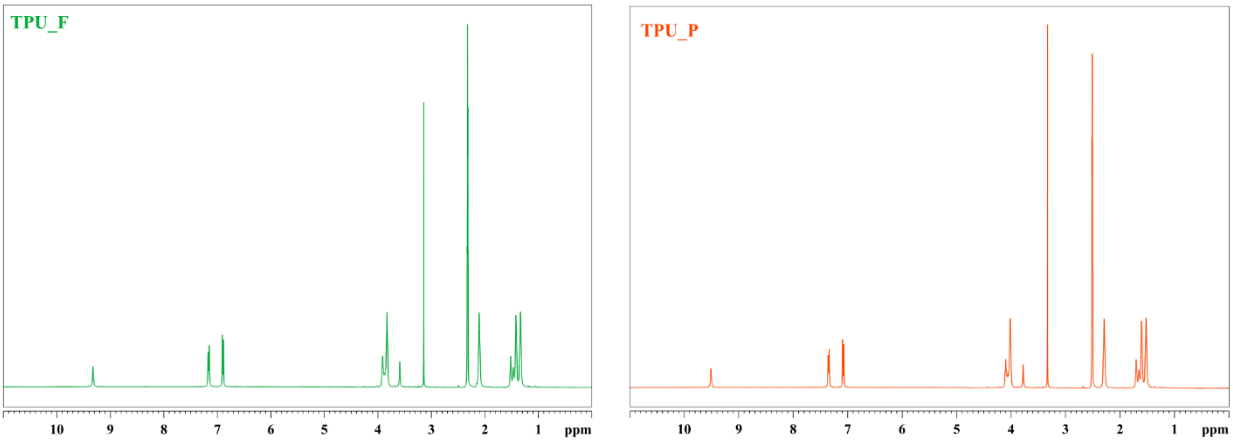

Figure S2. ^{1}H-NMR spectra of TPU_F and TPU_P.

Figure S2 shows the results of H-NMR measurements of TPU_F and TPU_P samples. H-NMR spectra allow the analysis of the chemical structure of the material based on the chemical shifts of the protons. According to literature studies on TPUs, a single peak above 9 ppm most likely corresponds to the protons derived from the urethane bond in the rigid TPU segments. While dublets present around 6.90 to 7.40 ppm correspond to methylene protons placed in aromatic rings of methylene diphenyl diisocyanate (MDI) used for TPU synthesis [1,2].

Long-term degradation in PBS

Table S6. Optical microscopy images of TPU_P samples obtained during long-term degradation study in 0.1 M PBS solution.

	Time of Degradation			
	Day 0	1 month	3 months	6 months
0.1M PBS	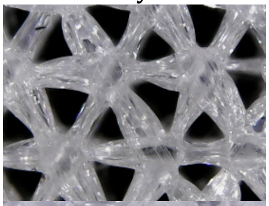 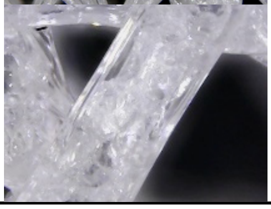	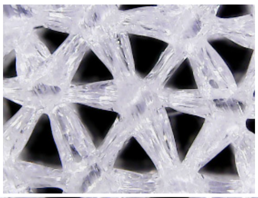 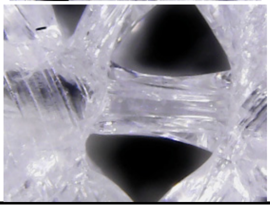	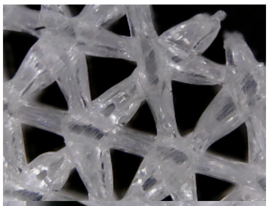 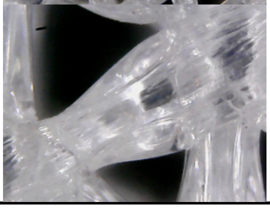	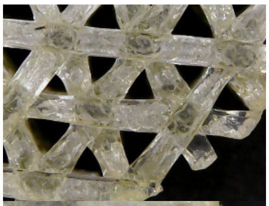 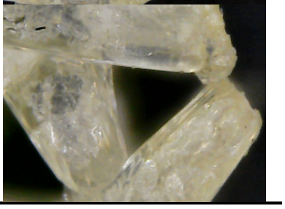

Studies of porous tissue structures (PTS)

Table S7. SEM images from the accelerated degradation studies of G25 porous tissue structure.

× 150	× 1000	× 5000

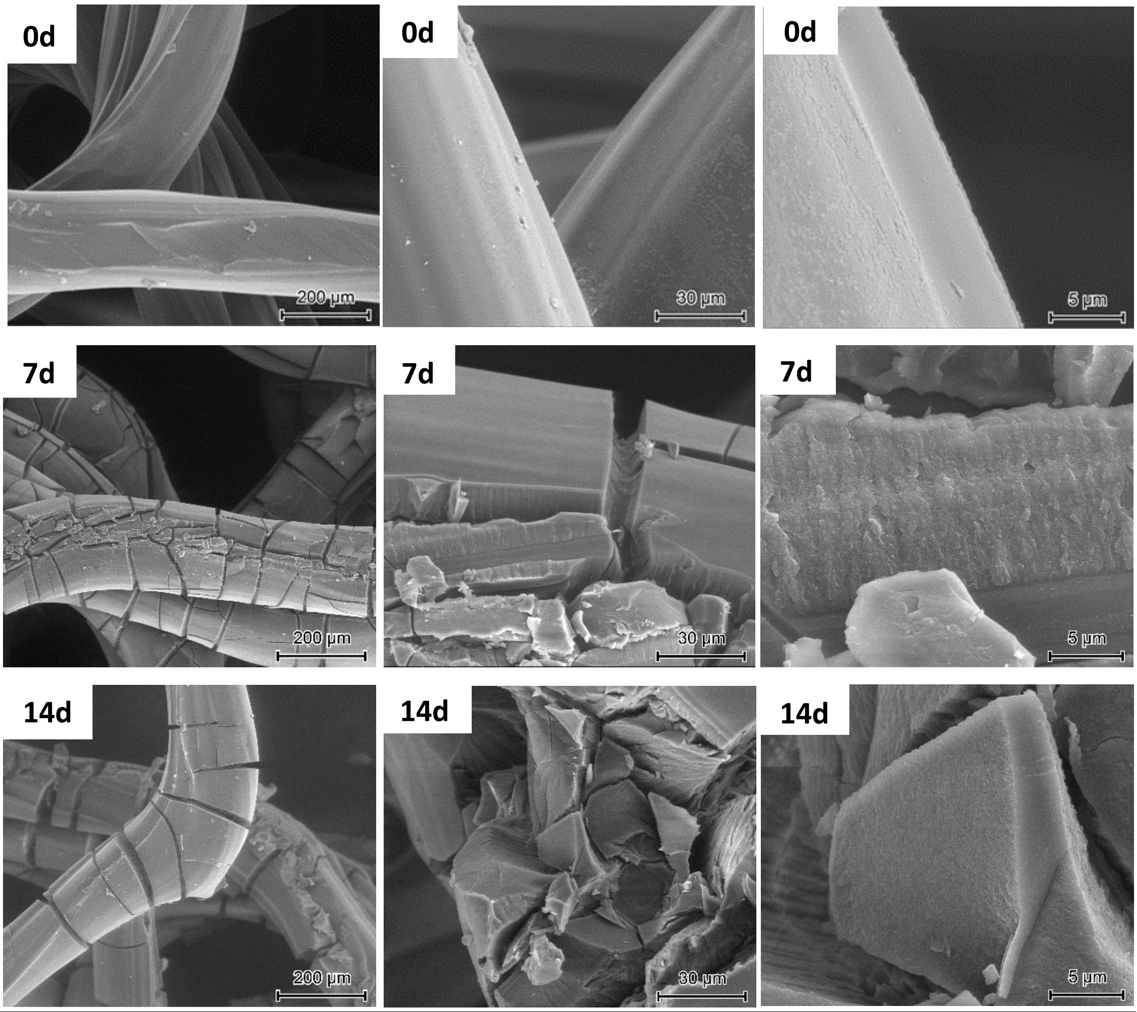

References

1. Parcheta, P.; Głowińska, E.; Datta, J. Effect of bio-based components on the chemical structure, thermal stability and mechanical properties of green thermoplastic polyurethane elastomers. *Eur. Polym. J.* **2020**, *123*, 109422, doi:10.1016/j.eurpolymj.2019.109422.
2. Choi, J.; Moon, D.S.; Jang, J.U.; Yin, W. Bin; Lee, B.; Lee, K.J. Synthesis of highly functionalized thermoplastic polyurethanes and their potential applications. *Polymer (Guildf).* **2017**, *116*, 287–294, doi:10.1016/j.polymer.2017.03.083.

© 2020 by the authors. Submitted for possible open access publication under the terms and conditions of the Creative Commons Attribution (CC BY) license (http://creativecommons.org/licenses/by/4.0/).
